# *N*-(4-Chloro­butanoyl)-*N*′-(2-fluoro­phen­yl)thio­urea

**DOI:** 10.1107/S1600536811024743

**Published:** 2011-06-30

**Authors:** M. Sukeri M. Yusof, Nur Farhana Embong, Eliyanti A. Othman, Bohari M. Yamin

**Affiliations:** a Department of Chemical Sciences, Faculty of Science and Technology, Universiti Malaysia Terengganu, 21030 Kuala Terengganu, Terengganu, Malaysia; b School of Chemical Sciences and Food Technology, Universiti Kebangsaan Malaysia, UKM 43500 Bangi Selangor, Malaysia

## Abstract

In the title compound, C_11_H_12_ClFN_2_OS, the asymmetric unit consists of two indenpendent mol­ecules. Both mol­ecules maintain a *trans*–*cis* configuration of the positions of the butanoyl and fluoro­phenyl groups with respect to the thiono group across their C—N bonds and are stabilized by classical intra­molecular N—H⋯O hydrogen bonds. In the crystal, inter­molecular N—H⋯O, C—H⋯S and N—H⋯S hydrogen bonds link the mol­ecules into infinite chains along the *c* axis.

## Related literature

For a related structure, see: Yamin *et al.* (2011 ▶). For standard bond lengths, see: Allen *et al.* (1987 ▶).
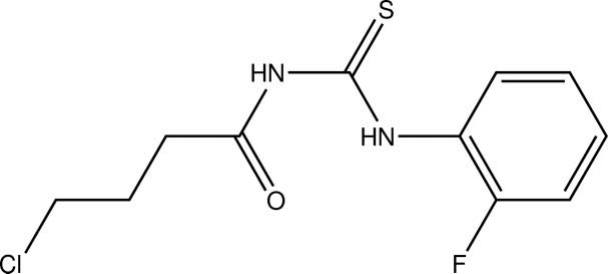


## Experimental

### 

#### Crystal data

C_11_H_12_ClFN_2_OS
*M* *_r_* = 274.75
Monoclinic, 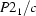

*a* = 14.818 (7) Å
*b* = 10.291 (5) Å
*c* = 18.201 (9) Å
β = 112.599 (12)°
*V* = 2562 (2) Å^3^
*Z* = 8
Mo *K*α radiation
μ = 0.46 mm^−1^
*T* = 298 K
0.50 × 0.22 × 0.07 mm

#### Data collection

Bruker SMART APEX CCD area-detector diffractometer
Absorption correction: multi-scan (*SADABS*; Bruker, 2000 ▶) *T*_min_ = 0.803, *T*_max_ = 0.969
13771 measured reflections
4501 independent reflections
2231 reflections with *I* > 2σ(*I*)
*R*_int_ = 0.119

#### Refinement

*R*[*F* ^2^ > 2σ(*F* ^2^)] = 0.063
*wR*(*F* ^2^) = 0.138
*S* = 0.89
4501 reflections
307 parameters
H-atom parameters constrained
Δρ_max_ = 0.34 e Å^−3^
Δρ_min_ = −0.30 e Å^−3^

### 

Data collection: *SMART* (Bruker, 2000 ▶); cell refinement: *SAINT* (Bruker, 2000 ▶); data reduction: *SAINT*; program(s) used to solve structure: *SHELXTL* (Sheldrick, 2008 ▶); program(s) used to refine structure: *SHELXTL*; molecular graphics: *SHELXTL*; software used to prepare material for publication: *SHELXTL*, *PARST* (Nardelli, 1995 ▶) and *PLATON* (Spek, 2009 ▶).

## Supplementary Material

Crystal structure: contains datablock(s) global, I. DOI: 10.1107/S1600536811024743/rk2280sup1.cif

Structure factors: contains datablock(s) I. DOI: 10.1107/S1600536811024743/rk2280Isup2.hkl

Supplementary material file. DOI: 10.1107/S1600536811024743/rk2280Isup3.cml

Additional supplementary materials:  crystallographic information; 3D view; checkCIF report

## Figures and Tables

**Table 1:** Hydrogen-bond geometry (Å, °)

*D*—H⋯*A*	*D*—H	H⋯*A*	*D*⋯*A*	*D*—H⋯*A*
N2—H2⋯O1	0.86	2.02	2.691 (5)	134
N2—H2⋯O2^i^	0.86	2.40	3.128 (5)	143
N4—H4⋯O2	0.86	2.02	2.676 (5)	133
N4—H4⋯O1^ii^	0.86	2.32	3.033 (5)	141
N3—H3⋯S1^iii^	0.86	2.59	3.447 (4)	176
N1—H1⋯S2^iii^	0.86	2.52	3.364 (4)	169
C14—H14*A*⋯S1^iii^	0.97	2.96	3.784 (5)	143
C14—H14*B*⋯S2^iv^	0.97	2.74	3.691 (5)	168

Symmetry codes: (i) 
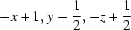
; (ii) 
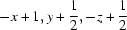
; (iii) 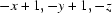; (iv) 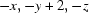.

**Fig. 1.**
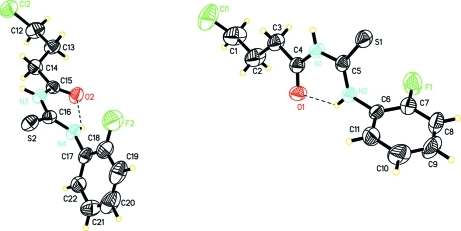
The molecular structure of title compound with the atom numbering scheme. Displacement ellipsoids are drawn at the 50% probability level. H atoms are presented as a small spheres of arbitrary radius. intramolecular H bonds are presented by dashed lines.

**Fig. 2.**
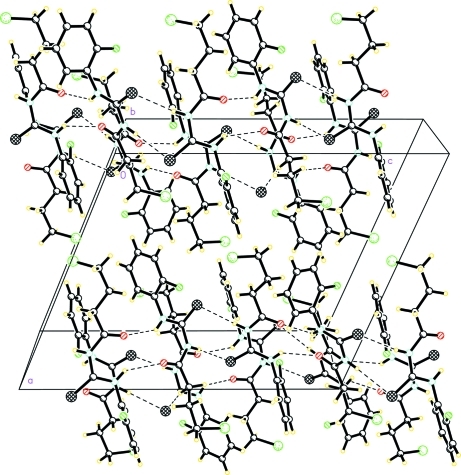
A packing diagram of title compound viewed down the a-axis. H bonds are shown by dashed lines.

## supplementary crystallographic information

### Comment

The title compound, is analogous to the previously reported *N*-(4-chlorobutanoyl)-*N*'-(phenyl)thiourea (Yamin *et al.*, 2011) except the fluoro atom is attached at the *ortho*-position of the phenyl ring. The asymmetric unit consists of two independent molecules (Fig. 1). The whole molecule is not planar. However, the thiourea N1/C5/S1/N2/C6, N3/C16/S2/N4/C17 fragments and the benzene rings, (C6–C11) and (C17–C22) are each planar with maximum deviation of 0.020 (3)Å for N4 atom from the least square plane. In each molecule, the benzene ring and thiourea moiety forms dihedral angle of 74.78 (19)° and 82.3 (2)°, respectively. The same dihedral angle in *N*-(4-chlorobutanoyl)-N'-(phenyl)thiourea are 72.98 (12)° and 81.47 (14)°, respectively. The bond lengths and angles are in normal ranges (Allen *et al.*, 1987) and comparable to those in the analog. Both molecules maintain their *trans*–*cis*-configuration with respect to the position of the butanoyl and fluorophenyl groups against the thiono C═S-group bond across the C—N bonds. Like in most of the carbonylthiourea derivatives, the classical intramolecular hydrogen bonds between the carbonyl oxygen atom and thioamide hydrogen atom, N2—H2···O1 and N4—H4···O2, in both molecules are present. In the crystal packing, the molecules are linked by N3—H3···S1^iii^ and N1—H1···S2^iii^; N2—H2···O2^i^ and N4—H4···O1^ii^; C14—H14A···S1^iii^ and C14—H14B···S2^iv^ intermolecular hydrogen bonds (symmetry codes as in Table 1) and form infinite chains along the *c*-axis (Fig. 2).

### Experimental

A solution of 4-chlorobutanoylisothiocyanate (1.25 g, 6.33 mmol) in 30 ml acetone was added into a flask containing 30 ml acetone solution of 2-floroaniline (0.71 g, 6.33 mmol. The mixture was refluxed for 1 h. Then, the solution was filtered-off and left to evaporate at room temperature. The colourless solid was obtained after one day of evaporation (yield 83%, m.p. 411.7 K–415.5 K)

### Refinement

All H atoms attached to C and N atoms were fixed geometrically and treated as riding with C—H = 0.93Å or 0.97Å (aromatic and methylene) and N—H = 0.86Å (amino) with *U*_iso_(H) = 1.2*U*_eq_(C, N).

### Crystal data

**Table:**

C_11_H_12_ClFN_2_OS	*F*(000) = 1136
*M**_r_* = 274.75	*D*_x_ = 1.425 Mg m^−^^3^
Monoclinic, *P*2_1_/*c*	Melting point = 411.7–415.5 K
Hall symbol: -P 2ybc	Mo *K*α radiation, λ = 0.71073 Å
*a* = 14.818 (7) Å	Cell parameters from 1404 reflections
*b* = 10.291 (5) Å	θ = 1.5–25.0°
*c* = 18.201 (9) Å	µ = 0.46 mm^−^^1^
β = 112.599 (12)°	*T* = 298 K
*V* = 2562 (2) Å^3^	Plate, colourless
*Z* = 8	0.50 × 0.22 × 0.07 mm

### Data collection

**Table:**

Bruker SMART APEX CCD area-detector diffractometer	4501 independent reflections
Radiation source: fine-focus sealed tube	2231 reflections with *I* > 2σ(*I*)
graphite	*R*_int_ = 0.119
Detector resolution: 83.66 pixels mm^-1^	θ_max_ = 25.0°, θ_min_ = 1.5°
ω scans	*h* = −17→16
Absorption correction: multi-scan (*SADABS*; Bruker, 2000)	*k* = −12→12
*T*_min_ = 0.803, *T*_max_ = 0.969	*l* = −13→21
13771 measured reflections	

### Refinement

**Table:**

Refinement on *F*^2^	Primary atom site location: structure-invariant direct methods
Least-squares matrix: full	Secondary atom site location: difference Fourier map
*R*[*F*^2^ > 2σ(*F*^2^)] = 0.063	Hydrogen site location: inferred from neighbouring sites
*wR*(*F*^2^) = 0.138	H-atom parameters constrained
*S* = 0.89	*w* = 1/[σ^2^(*F*_o_^2^) + (0.0451*P*)^2^] where *P* = (*F*_o_^2^ + 2*F*_c_^2^)/3
4501 reflections	(Δ/σ)_max_ < 0.001
307 parameters	Δρ_max_ = 0.34 e Å^−^^3^
0 restraints	Δρ_min_ = −0.30 e Å^−^^3^

### Special details

**Table:**

Geometry. All s.u.'s (except the s.u. in the dihedral angle between two l.s. planes) are estimated using the full covariance matrix. The cell s.u.'s are taken into account individually in the estimation of s.u.'s in distances, angles and torsion angles; correlations between s.u.'s in cell parameters are only used when they are defined by crystal symmetry. An approximate (isotropic) treatment of cell s.u.'s is used for estimating s.u.'s involving l.s. planes.
Refinement. Refinement of *F*^2^ against ALL reflections. The weighted *R*-factor *wR* and goodness of fit *S* are based on *F*^2^, conventional *R*-factors *R* are based on *F*, with *F* set to zero for negative *F*^2^. The threshold expression of *F*^2^ > σ(*F*^2^) is used only for calculating *R*-factors(gt) *etc*. and is not relevant to the choice of reflections for refinement. *R*-factors based on *F*^2^ are statistically about twice as large as those based on *F*, and *R*-factors based on ALL data will be even larger.

### Fractional atomic coordinates and isotropic or equivalent isotropic displacement parameters (Å^2^)

**Table:**

	*x*	*y*	*z*	*U*_iso_*/*U*_eq_	
Cl1	0.49078 (11)	0.21242 (19)	0.00799 (10)	0.1269 (7)	
F1	1.0669 (2)	−0.1688 (3)	0.35144 (16)	0.0795 (9)	
F2	0.3894 (2)	0.7354 (3)	0.12270 (17)	0.0868 (9)	
S1	1.02276 (8)	−0.03047 (12)	0.17159 (6)	0.0509 (3)	
S2	0.24427 (8)	1.01331 (12)	0.02342 (6)	0.0502 (3)	
Cl2	−0.23288 (9)	0.68710 (13)	−0.21630 (7)	0.0804 (5)	
O1	0.80228 (19)	0.1449 (3)	0.26210 (18)	0.0562 (9)	
O2	0.0637 (2)	0.6917 (3)	0.07106 (17)	0.0552 (9)	
N1	0.8543 (2)	0.0465 (3)	0.17333 (18)	0.0424 (9)	
H1	0.8335	0.0212	0.1246	0.051*	
N2	0.9869 (2)	0.0693 (3)	0.29214 (19)	0.0428 (9)	
H2	0.9467	0.1033	0.3103	0.051*	
N3	0.0918 (2)	0.8641 (3)	0.00426 (18)	0.0421 (9)	
H3	0.0658	0.9086	−0.0388	0.051*	
N4	0.2217 (2)	0.8449 (3)	0.12431 (18)	0.0447 (9)	
H4	0.1883	0.7860	0.1360	0.054*	
C1	0.5036 (3)	0.1269 (6)	0.0958 (3)	0.0858 (19)	
H1A	0.4571	0.1608	0.1167	0.103*	
H1B	0.4885	0.0359	0.0831	0.103*	
C2	0.6047 (3)	0.1386 (5)	0.1580 (3)	0.0718 (16)	
H2A	0.6195	0.2299	0.1700	0.086*	
H2B	0.6065	0.0966	0.2063	0.086*	
C3	0.6818 (3)	0.0806 (5)	0.1350 (2)	0.0547 (13)	
H3A	0.6790	0.1208	0.0860	0.066*	
H3B	0.6682	−0.0113	0.1246	0.066*	
C4	0.7839 (3)	0.0961 (4)	0.1974 (3)	0.0453 (11)	
C5	0.9539 (3)	0.0312 (4)	0.2162 (2)	0.0410 (11)	
C6	1.0875 (3)	0.0553 (4)	0.3442 (2)	0.0407 (11)	
C7	1.1249 (3)	−0.0633 (5)	0.3737 (3)	0.0522 (12)	
C8	1.2206 (4)	−0.0794 (6)	0.4252 (3)	0.0739 (16)	
H8	1.2448	−0.1613	0.4445	0.089*	
C9	1.2793 (4)	0.0285 (7)	0.4472 (3)	0.0822 (19)	
H9	1.3443	0.0198	0.4816	0.099*	
C10	1.2431 (3)	0.1484 (6)	0.4192 (3)	0.0786 (17)	
H10	1.2835	0.2210	0.4346	0.094*	
C11	1.1459 (3)	0.1625 (5)	0.3675 (3)	0.0610 (14)	
H11	1.1209	0.2444	0.3489	0.073*	
C12	−0.2143 (3)	0.6210 (5)	−0.1206 (3)	0.0691 (15)	
H12A	−0.2532	0.6696	−0.0977	0.083*	
H12B	−0.2369	0.5316	−0.1269	0.083*	
C13	−0.1093 (3)	0.6249 (4)	−0.0646 (2)	0.0530 (13)	
H13A	−0.0713	0.5723	−0.0865	0.064*	
H13B	−0.1036	0.5862	−0.0145	0.064*	
C14	−0.0659 (3)	0.7590 (4)	−0.0487 (2)	0.0490 (12)	
H14A	−0.0639	0.7934	−0.0977	0.059*	
H14B	−0.1086	0.8147	−0.0334	0.059*	
C15	0.0351 (3)	0.7652 (4)	0.0149 (2)	0.0418 (11)	
C16	0.1846 (3)	0.9006 (4)	0.0537 (2)	0.0386 (10)	
C17	0.3156 (3)	0.8794 (4)	0.1821 (2)	0.0453 (12)	
C18	0.3983 (3)	0.8244 (5)	0.1795 (3)	0.0566 (13)	
C19	0.4902 (3)	0.8541 (6)	0.2342 (3)	0.0781 (17)	
H19	0.5457	0.8151	0.2319	0.094*	
C20	0.4970 (4)	0.9431 (7)	0.2921 (4)	0.091 (2)	
H20	0.5584	0.9657	0.3291	0.109*	
C21	0.4160 (4)	0.9992 (5)	0.2966 (3)	0.0777 (17)	
H21	0.4221	1.0588	0.3366	0.093*	
C22	0.3243 (3)	0.9664 (4)	0.2409 (3)	0.0571 (13)	
H22	0.2686	1.0039	0.2438	0.069*	

### Atomic displacement parameters (Å^2^)

**Table:**

	*U*^11^	*U*^22^	*U*^33^	*U*^12^	*U*^13^	*U*^23^
Cl1	0.0706 (11)	0.1612 (18)	0.1063 (14)	0.0426 (11)	−0.0131 (9)	0.0101 (12)
F1	0.077 (2)	0.0548 (19)	0.098 (2)	0.0034 (15)	0.0232 (16)	0.0093 (16)
F2	0.068 (2)	0.111 (3)	0.081 (2)	0.0213 (17)	0.0283 (16)	−0.0051 (19)
S1	0.0373 (6)	0.0695 (9)	0.0458 (7)	0.0083 (6)	0.0157 (5)	−0.0007 (6)
S2	0.0383 (6)	0.0665 (9)	0.0432 (7)	−0.0099 (6)	0.0128 (5)	0.0030 (6)
Cl2	0.0695 (9)	0.0838 (10)	0.0632 (9)	−0.0010 (7)	−0.0020 (7)	−0.0005 (7)
O1	0.0393 (18)	0.072 (2)	0.051 (2)	0.0063 (15)	0.0105 (15)	−0.0195 (17)
O2	0.0491 (19)	0.050 (2)	0.053 (2)	−0.0104 (15)	0.0043 (15)	0.0103 (16)
N1	0.0292 (19)	0.059 (2)	0.035 (2)	0.0022 (17)	0.0072 (16)	−0.0047 (16)
N2	0.0299 (19)	0.052 (2)	0.041 (2)	0.0042 (16)	0.0075 (16)	−0.0039 (17)
N3	0.032 (2)	0.052 (2)	0.036 (2)	−0.0023 (17)	0.0056 (16)	0.0003 (16)
N4	0.031 (2)	0.054 (2)	0.043 (2)	−0.0076 (16)	0.0067 (16)	0.0077 (17)
C1	0.030 (3)	0.135 (5)	0.086 (4)	−0.007 (3)	0.014 (3)	−0.035 (4)
C2	0.037 (3)	0.110 (5)	0.065 (3)	−0.002 (3)	0.017 (2)	−0.014 (3)
C3	0.031 (2)	0.078 (4)	0.051 (3)	0.006 (2)	0.011 (2)	−0.003 (2)
C4	0.037 (3)	0.051 (3)	0.045 (3)	0.003 (2)	0.013 (2)	−0.001 (2)
C5	0.033 (2)	0.040 (3)	0.045 (3)	−0.0013 (19)	0.009 (2)	0.003 (2)
C6	0.032 (2)	0.053 (3)	0.033 (2)	0.004 (2)	0.0073 (18)	−0.004 (2)
C7	0.046 (3)	0.055 (3)	0.051 (3)	0.007 (3)	0.013 (2)	−0.002 (2)
C8	0.058 (4)	0.089 (4)	0.061 (3)	0.032 (3)	0.007 (3)	0.008 (3)
C9	0.034 (3)	0.133 (6)	0.063 (4)	0.022 (3)	0.001 (3)	−0.007 (4)
C10	0.036 (3)	0.100 (5)	0.089 (4)	−0.013 (3)	0.011 (3)	−0.026 (3)
C11	0.047 (3)	0.055 (3)	0.070 (3)	0.003 (2)	0.010 (2)	−0.007 (3)
C12	0.050 (3)	0.082 (4)	0.068 (3)	−0.022 (3)	0.015 (3)	−0.010 (3)
C13	0.046 (3)	0.059 (3)	0.048 (3)	−0.012 (2)	0.011 (2)	−0.004 (2)
C14	0.032 (2)	0.052 (3)	0.060 (3)	0.003 (2)	0.014 (2)	−0.004 (2)
C15	0.032 (2)	0.042 (3)	0.045 (3)	0.000 (2)	0.008 (2)	−0.007 (2)
C16	0.028 (2)	0.048 (3)	0.038 (3)	−0.0008 (19)	0.0109 (19)	−0.003 (2)
C17	0.031 (3)	0.053 (3)	0.043 (3)	−0.005 (2)	0.005 (2)	0.010 (2)
C18	0.045 (3)	0.069 (4)	0.053 (3)	0.000 (3)	0.016 (2)	0.005 (3)
C19	0.037 (3)	0.104 (5)	0.082 (4)	0.006 (3)	0.011 (3)	0.025 (4)
C20	0.047 (4)	0.105 (5)	0.089 (5)	−0.022 (4)	−0.007 (3)	0.022 (4)
C21	0.068 (4)	0.077 (4)	0.068 (4)	−0.016 (3)	0.005 (3)	−0.013 (3)
C22	0.050 (3)	0.053 (3)	0.058 (3)	0.001 (2)	0.009 (2)	0.000 (3)

### Geometric parameters (Å, °)

**Table:**

Cl1—C1	1.769 (6)	C6—C7	1.361 (6)
F1—C7	1.347 (5)	C6—C11	1.365 (5)
F2—C18	1.350 (5)	C7—C8	1.377 (6)
S1—C5	1.654 (4)	C8—C9	1.372 (7)
S2—C16	1.675 (4)	C8—H8	0.9300
Cl2—C12	1.791 (5)	C9—C10	1.364 (7)
O1—C4	1.212 (4)	C9—H9	0.9300
O2—C15	1.210 (4)	C10—C11	1.393 (6)
N1—C4	1.374 (5)	C10—H10	0.9300
N1—C5	1.388 (4)	C11—H11	0.9300
N1—H1	0.8600	C12—C13	1.498 (5)
N2—C5	1.336 (5)	C12—H12A	0.9700
N2—C6	1.433 (5)	C12—H12B	0.9700
N2—H2	0.8600	C13—C14	1.502 (5)
N3—C16	1.375 (4)	C13—H13A	0.9700
N3—C15	1.380 (5)	C13—H13B	0.9700
N3—H3	0.8600	C14—C15	1.501 (5)
N4—C16	1.318 (4)	C14—H14A	0.9700
N4—C17	1.430 (5)	C14—H14B	0.9700
N4—H4	0.8600	C17—C22	1.363 (6)
C1—C2	1.496 (5)	C17—C18	1.366 (6)
C1—H1A	0.9700	C18—C19	1.377 (6)
C1—H1B	0.9700	C19—C20	1.369 (7)
C2—C3	1.485 (6)	C19—H19	0.9300
C2—H2A	0.9700	C20—C21	1.363 (8)
C2—H2B	0.9700	C20—H20	0.9300
C3—C4	1.511 (5)	C21—C22	1.389 (6)
C3—H3A	0.9700	C21—H21	0.9300
C3—H3B	0.9700	C22—H22	0.9300
			
C4—N1—C5	129.5 (4)	C9—C10—C11	120.2 (5)
C4—N1—H1	115.2	C9—C10—H10	119.9
C5—N1—H1	115.2	C11—C10—H10	119.9
C5—N2—C6	121.9 (4)	C6—C11—C10	119.5 (5)
C5—N2—H2	119.0	C6—C11—H11	120.2
C6—N2—H2	119.0	C10—C11—H11	120.2
C16—N3—C15	128.2 (3)	C13—C12—Cl2	112.4 (4)
C16—N3—H3	115.9	C13—C12—H12A	109.1
C15—N3—H3	115.9	Cl2—C12—H12A	109.1
C16—N4—C17	122.1 (3)	C13—C12—H12B	109.1
C16—N4—H4	118.9	Cl2—C12—H12B	109.1
C17—N4—H4	118.9	H12A—C12—H12B	107.8
C2—C1—Cl1	111.9 (4)	C12—C13—C14	114.3 (4)
C2—C1—H1A	109.2	C12—C13—H13A	108.7
Cl1—C1—H1A	109.2	C14—C13—H13A	108.7
C2—C1—H1B	109.2	C12—C13—H13B	108.7
Cl1—C1—H1B	109.2	C14—C13—H13B	108.7
H1A—C1—H1B	107.9	H13A—C13—H13B	107.6
C3—C2—C1	114.2 (4)	C15—C14—C13	114.4 (3)
C3—C2—H2A	108.7	C15—C14—H14A	108.7
C1—C2—H2A	108.7	C13—C14—H14A	108.7
C3—C2—H2B	108.7	C15—C14—H14B	108.7
C1—C2—H2B	108.7	C13—C14—H14B	108.7
H2A—C2—H2B	107.6	H14A—C14—H14B	107.6
C2—C3—C4	113.7 (4)	O2—C15—N3	123.0 (4)
C2—C3—H3A	108.8	O2—C15—C14	123.2 (4)
C4—C3—H3A	108.8	N3—C15—C14	113.8 (4)
C2—C3—H3B	108.8	N4—C16—N3	117.2 (4)
C4—C3—H3B	108.8	N4—C16—S2	123.2 (3)
H3A—C3—H3B	107.7	N3—C16—S2	119.7 (3)
O1—C4—N1	123.3 (4)	C22—C17—C18	119.0 (4)
O1—C4—C3	124.0 (4)	C22—C17—N4	120.8 (4)
N1—C4—C3	112.6 (4)	C18—C17—N4	120.3 (4)
N2—C5—N1	115.8 (4)	F2—C18—C17	118.9 (4)
N2—C5—S1	124.8 (3)	F2—C18—C19	118.9 (5)
N1—C5—S1	119.4 (3)	C17—C18—C19	122.2 (5)
C7—C6—C11	119.3 (4)	C20—C19—C18	117.7 (5)
C7—C6—N2	120.9 (4)	C20—C19—H19	121.2
C11—C6—N2	119.8 (4)	C18—C19—H19	121.2
F1—C7—C6	119.2 (4)	C21—C20—C19	121.6 (5)
F1—C7—C8	118.6 (5)	C21—C20—H20	119.2
C6—C7—C8	122.2 (5)	C19—C20—H20	119.2
C9—C8—C7	118.2 (5)	C20—C21—C22	119.4 (5)
C9—C8—H8	120.9	C20—C21—H21	120.3
C7—C8—H8	120.9	C22—C21—H21	120.3
C10—C9—C8	120.6 (5)	C17—C22—C21	120.2 (5)
C10—C9—H9	119.7	C17—C22—H22	119.9
C8—C9—H9	119.7	C21—C22—H22	119.9

### Hydrogen-bond geometry (Å, °)

**Table:**

*D*—H···*A*	*D*—H	H···*A*	*D*···*A*	*D*—H···*A*
N2—H2···O1	0.86	2.02	2.691 (5)	134
N2—H2···O2^i^	0.86	2.40	3.128 (5)	143
N4—H4···O2	0.86	2.02	2.676 (5)	133
N4—H4···O1^ii^	0.86	2.32	3.033 (5)	141
N3—H3···S1^iii^	0.86	2.59	3.447 (4)	176
N1—H1···S2^iii^	0.86	2.52	3.364 (4)	169
C3—H3A···Cl1	0.97	2.76	3.189 (5)	107
C14—H14A···Cl2	0.97	2.82	3.190 (4)	103
C14—H14A···S1^iii^	0.97	2.96	3.784 (5)	143
C14—H14B···S2^iv^	0.97	2.74	3.691 (5)	168

Symmetry codes:  (i) −*x*+1, *y*−1/2, −*z*+1/2; (ii) −*x*+1, *y*+1/2, −*z*+1/2; (iii) −*x*+1, −*y*+1, −*z*; (iv) −*x*, −*y*+2, −*z*.
